# In Vitro Evaluation of Necrostatin-1 Microparticles for Limiting Stress-induced Cell Death and Preserving Islet Function

**DOI:** 10.1097/TXD.0000000000001945

**Published:** 2026-04-23

**Authors:** Nerea Cuesta-Gomez, Pulkit Kumar, Purushothaman Kuppan, Joy Paramor, Abdullah Saleh, Gregory S. Korbutt, Andrew R. Pepper

**Affiliations:** 1 Department of Surgery, University of Alberta, Edmonton, AB, Canada.; 2 Alberta Diabetes Institute, University of Alberta, Edmonton, AB, Canada.

## Abstract

**Background.:**

Islet transplantation offers a promising therapy for type 1 diabetes, but early β-cell loss due to stress-induced cell death limits graft survival and function. Although apoptosis has been extensively studied, necroptosis, a regulated form of necrotic cell death, remains an underexplored contributor to β-cell dysfunction. Herein, we evaluated sustained necrostatin-1 (Nec-1) delivery via poly(lactic-co-glycolic acid) (PLGA) microparticles (MPs) to protect islets from stress-induced damage in vitro.

**Methods.:**

Nec-1-loaded PLGA MPs were synthesized by single-emulsion solvent evaporation and characterized by scanning electron microscopy and high-performance liquid chromatography for size, morphology, and drug encapsulation. Mouse islets were coincubated with Nec-1 or empty MPs for 24 h, followed by induction of endoplasmic reticulum stress with thapsigargin. Glucose-stimulated insulin secretion and cytokine release were quantified to assess β-cell function and inflammatory response.

**Results.:**

Nec-1 MPs were spherical with a median diameter of 10.34 μm (interquartile range [IQR], 6.55–15.17 μm) and an encapsulation efficiency of 36.2% (IQR, 31.1%–40.0%). Release studies demonstrated an initial burst of 24.8% (IQR, 21.8%–30.3%) by day 3, followed by sustained delivery up to 53.6% (IQR, 52.3%–57.4%) by day 14, with minimal impact on media pH. Under thapsigargin-induced stress, Nec-1 MP-treated mouse islets maintained glucose-stimulated insulin secretion, with insulin release increasing from 2.63% (IQR, 1.42%–5.74%) to 8.63% (IQR, 7.97%–14.42%, *P* = 0.0006) compared with 3.36% (IQR, 0.53%–9.08%) in stressed controls (*P = *0.0117). The Stimulation Index was similarly preserved (3.23; IQR, 2.51–4.77 versus 1.18; IQR, 0.65–1.62; *P = *0.0002). Nec-1 MPs partially attenuated IL-12p70 secretion (103.3 pg/mL; IQR, 67.7–162.4 versus 217.1 pg/mL; IQR, 86.2–367.6; *P* = 0.0381) while maintaining low levels of other proinflammatory cytokines.

**Conclusions.:**

Nec-1-loaded PLGA MPs provide sustained, localized protection of β-cells from necroptotic and inflammatory stress in vitro. Given the sustained and targeted drug delivery, these findings provide a foundation for future in vivo translation to provide graft-localized therapeutic intervention and improve islet survival, engraftment, and long-term function.

## INTRODUCTION

Islet transplantation (ITx) offers a physiological means to restore endogenous insulin production in patients with type 1 diabetes; however, widespread clinical application remains limited by poor long-term graft survival.^1,2^ Following transplantation, islets are exposed to a hostile microenvironment characterized by hypoxia, inflammation, and oxidative stress.^3,4^ As a result, up to 70% of transplanted islets fail to engraft within the first few days posttransplantation.^5-9^ This early loss of viable islet mass severely compromises graft function and triggers recurrent autoimmunity and alloimmune rejection by increasing the release of damage-associated molecular patterns and donor antigens.^10^ Consequently, strategies to improve islet engraftment and protect against early cell death are essential to enhance long-term transplant outcomes.

Our laboratory and others have identified regulated cell death pathways, particularly apoptosis and necrosis, as major drivers of early islet loss.^11^ In ongoing studies, we have observed that pretransplant inhibition of necroptosis with necrostatin-1 (Nec-1) for 24 h markedly improves islet survival and graft outcomes, consistent with other studies implicating blockade of death receptor-mediated and caspase-independent pathways in preserving islet integrity.^11-15^ These findings provide strong in vivo evidence that pharmacological inhibition of necroptosis protects islets during the critical peritransplant period.

Systemic delivery of immunosuppressive agents or cell death inhibitors carries significant side effects and toxicity, limiting long-term use.^16-20^ To overcome these limitations, our group has developed microparticle (MP)-based drug delivery systems for localized, sustained release of immunomodulatory agents at the transplant site. Specifically, we use poly(lactic-co-glycolic acid) (PLGA), a Food and Drug Administration–authorized biodegradable and biocompatible polymer approved for numerous biomedical applications, including drug delivery, to fabricate these MPs, enabling controlled and sustained release of therapeutics directly at the graft site.^21^ Using this platform, we have shown that localized delivery of cyclosporine A and dexamethasone effectively protects transplanted islets, improves engraftment, and supports long-term graft function while minimizing systemic exposure and toxicity.^22,23^ Other groups have similarly demonstrated that site-specific immunomodulation can promote graft tolerance and protection, for instance, via local presentation of Fas ligand (FasL) on biomaterials, cytokine-releasing scaffolds, and apoptotic cell-mimetic systems, which modulate immune cell infiltration and reduce alloreactivity in transplantation models.^24-27^ Collectively, these approaches highlight the potential of engineering the local graft environment to drive immune privilege without systemic immunosuppression.

Herein, we hypothesize that localized delivery of Nec-1 at the graft site can further enhance islet survival by reducing early cell death and promoting engraftment. To achieve this, we leverage our established MP platform as a vehicle for sustained, site-specific Nec-1 release alongside transplanted islets.^22,23^ As a first step, the present study investigates the in vitro effects of Nec-1 MPs on islet viability and function under transplant-relevant stress conditions. By defining the functional impact of Nec-1 MP treatment in vitro, this work establishes the foundation for using Nec-1-loaded MPs as a novel therapeutic strategy to improve islet engraftment and long-term graft success in vivo.

## MATERIALS AND METHODS

### Formulation and Characterization of Nec-1-eluting MPs

PLGA MPs were synthesized using an oil-in-water single emulsion solvent evaporation method. PLGA (200 mg; Sigma-Aldrich) and Nec-1 (2 mg, Sigma-Aldrich) were dissolved in 4 mL of dichloromethane (Sigma-Aldrich) and emulsified in 10 mL of 4% polyvinyl alcohol (Sigma-Aldrich) under vigorous magnetic stirring (~1000 rpm) at room temperature for 5 min.

The emulsion was transferred dropwise into 200 mL of 2% polyvinyl alcohol, stirred at 1000 rpm for 1 h, and stored at 4 °C overnight. The following day, MPs were collected by centrifugation (3000 rpm, 5 min, 4 °C), washed 3 times with Milli-Q water, freeze-dried, and stored at 4 °C. Empty (drug-free) MPs were prepared identically without Nec-1 and used as controls.

Particle morphology and size were assessed by scanning electron microscopy (ZEISS EVO 10). Lyophilized MPs were spread onto carbon tape, gold sputter-coated, and imaged. Nec-1 encapsulation efficiency was quantified by reverse-phase high-performance liquid chromatography (HPLC; Agilent Technologies). Lyophilized MPs (n = 6) were dissolved in an acetonitrile: methanol (8:2, v/v), centrifuged, and 10 µL of supernatant was injected into a C18 column (Agilent XDB-C18 column, 4.6 × 100 mm, 5 µm). Nec-1 was detected at 280 nm using a linear gradient (25%–95% acetonitrile containing 0.1% trifluoroacetic acid, 0.75 mL/min). Quantification was performed using standard curves (1–100 µg/mL). All experiments used MPs from the same batch; MP mass (1–4 mg) refers to the total amount of Nec-1-loaded MPs added per assay.

In vitro Nec-1 release was assessed by incubating 4 mg of Nec-1 in 1 mL of PBS (Thermo Fisher Scientific) at 37 °C. 100 µL aliquots were collected and stored at −20 °C for subsequent HPLC analysis.

In vivo Nec-1 release was characterized by resuspending 4 mg of Nec-1 MPs in 14 µL of 10 mg/mL rat tail collagen and transplanting them under the kidney capsule of B6.129S7-*Rag1*^*tm1Mom*^ (B6/Rag^−/−^) mice (Jackson Laboratory). Grafts were procured at 1, 3, 7, 14, and 21 d posttransplant (n = 3 per time point). Tissues were frozen and homogenized in acetonitrile:methanol (8:2, v/v), centrifuged (2000 relative centrifugal force, 5 min, 4 °C), and analyzed by HPLC.

### Murine Islet Isolation and Culture

All animal studies were approved by the University of Alberta Animal Care and Use Committee (AUP00003230, AUP00002977) and conducted in accordance with Canadian Council on Animal Care guidelines. Male BALB/c mice (8–12 wk old, 22–27 g; Jackson Laboratory) were used as islet donors.^28^ Islets were cultured in Connaught Medical Research Laboratories (CMRL)-1066 (Corning) supplemented with 2 mM GlutaMAX, 20 mM (4-[2-hydroxyethyl]-1-piperazineethanesulfonic acid), 100 U/mL penicillin, 100 μg/mL streptomycin, 5 mM sodium pyruvate, 10 mM nicotinamide, and 10% v/v heat-inactivated fetal bovine serum.

Islets were coincubated with 4 mg of Nec-1 MPs or empty MPs for 24 h, using 40 μm mesh inserts with MPs dispersed in 9 mL of medium per well. Based on particle size (median diameter 10.34 μm) and PLGA density (1.25 g/cm^3^), 4 mg of MPs corresponds to ~5.51 × 106 particles (pellet volume ~5 µL). Endoplasmic reticulum (ER) stress was induced by adding 5 μM of thapsigargin (Thp; Thermo Fisher Scientific) for an additional 24 h at 37 °C with 5% CO_2_. Supernatants were collected for cytokine analysis.

### Immunohistochemistry

Immunostaining was performed as previously described.^29^ Antigen retrieval was performed using Tris-EDTA buffer (pH 9.0). Sections were incubated overnight at 4 °C with antibodies against phosphorylated mixed lineage kinase domain-like protein (pMLKL; Thermo Fisher Scientific, Catalog No. PA5-105678; 1:100), cleaved caspase-3 (CC3; Cell Signaling Technology, Catalog No. 9664; 1:100), and insulin (guinea pig anti-insulin; DAKO, Catalog No. IR002; 1:5), followed by appropriate secondary antibodies for 1 h at room temperature. Slides were visualized using a Zeiss Observer Z1 and analyzed using QuPath.

### Glucose-stimulated Insulin Secretion Assay

Twenty-four hours after Thp incubation, 30 islets per condition (in triplicate) were washed and preincubated for 1 h in low-glucose Roswell Park Memorial Institute medium (RPMI)-1640 (2.8 mmol/L). Islets were sequentially incubated in low- and high-glucose RPMI (16.7 mmol/L) for 1 h each, with supernatants collected after each incubation. Insulin secretion was normalized to total insulin content, determined after acid-ethanol lysis, and quantified using a mouse/rat insulin assay (Meso Scale Discovery).

### Multiplex Cytokine Assay

Cytokine levels (tumor necrosis factor alpha [TNF-α], interleukin [IL]-6, IL-1β, keratinocyte chemoattractant/growth-regulated oncogene (KC GRO), IL-4, IL-5, IL-2, IL-12p70, IL-10, and interferon-gamma [IFN-γ]) were quantified using the V-PLEX Proinflammatory Panel 1 Mouse Kit (Meso Scale Discovery) according to the manufacturer’s instructions.

### Statistical Analysis

Data variability and distribution were assessed to confirm nonparametric test assumptions. Between-group comparisons were performed using the Mann-Whitney *U* test for 2 groups or the Kruskal-Wallis test for >2 groups, with Dunn’s post hoc test for multiple comparisons where applicable. Continuous data are presented as median and interquartile range (IQR); categorical data as counts and percentages. Statistical analyses were performed using GraphPad Prism version 9.3.1.

## RESULTS

### PLGA-mediated Encapsulation Enables Nec-1 MP Formation

**FIGURE 1.**
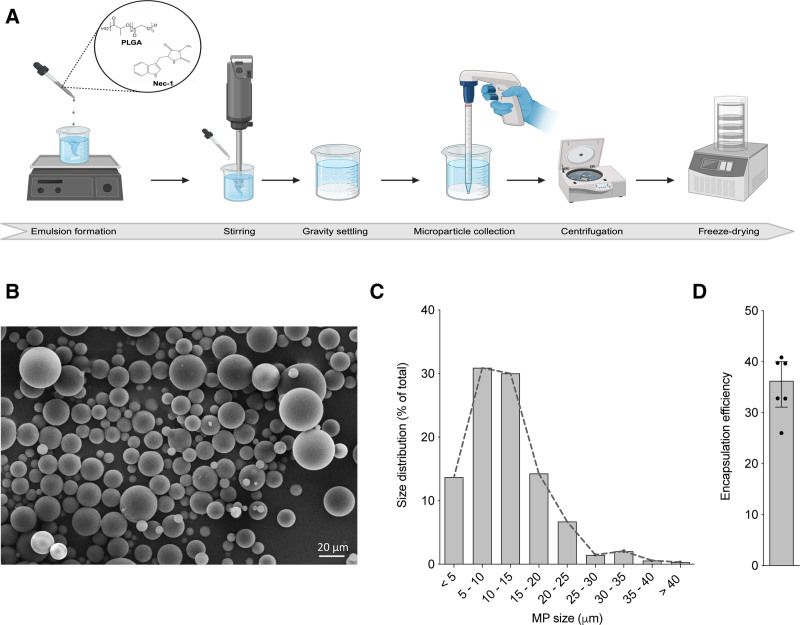
PLGA-mediated encapsulation enables Nec-1 MP formation. A, Schematic illustration of the single emulsion method used to synthesize Nec-1 MPs. PLGA and Nec-1 were dissolved in DCM and emulsified in PVA solution under high-speed mechanical stirring, followed by centrifugation and freeze-drying to obtain MPs. B, Representative SEM image of Nec-1 MPs, showing spherical morphology and smooth surface texture. C, Particle size distribution histogram of Nec-1 MPs. D, Encapsulation efficiency of Nec-1 in 6 independent MP batches. DCM, dichloromethane; MP, microparticles; Nec-1, necrostatin-1; PLGA, poly(lactic-co-glycolic acid); PVA, polyvinyl alcohol; SEM, scanning electron microscopy. Created in BioRender.

Nec-1 eluting MPs were synthesized using a single emulsion solvent evaporation technique (Figure 1A). Scanning electron microscopy revealed spherical, uniformly shaped particles with smooth surfaces (Figure 1B). Particle size distribution showed a right-skewed profile, with most particles ranging from 5 to 15 μm and a small fraction extending up to 52 μm. The median particle diameter was 10.34 μm (IQR, 6.55–15.17 μm; Figure 1C).

The total Nec-1 content in 1 mg of lyophilized PLGA MPs was 3.59 μg (IQR, 3.07–3.96 μg), corresponding to an encapsulation efficiency of 36.22% (IQR, 31.08%–40.02%; Figure 1D). In all experiments, the terms 1, 2, or 4 mg Nec-1 MPs refer to the amount of Nec-1-loaded MPs used in the assay. Because all MPs were prepared using the same protocol, each batch contains a consistent Nec-1 content per particle. These results confirm that PLGA encapsulation produces Nec-1 MPs of consistent size and drug loading suitable for in vitro islet studies.

### Nec-1 MPs Exhibit Sustained Drug Release With Minimal pH Impact

To assess the suitability of Nec-1 MPs for colocalization with islets, we evaluated their influence on culture media pH over time. PLGA degradation can produce acidic byproducts such as lactic acid and glycolic acid, which can acidify the local environment and compromise islet survival and function, particularly in confined transplant niches with limited buffering capacity. Nec-1 MPs were incubated at 3 concentrations (1, 2, or 4 mg) in either PBS or CMRL media for 21 d, alongside a no-MP control.

**FIGURE 2.**
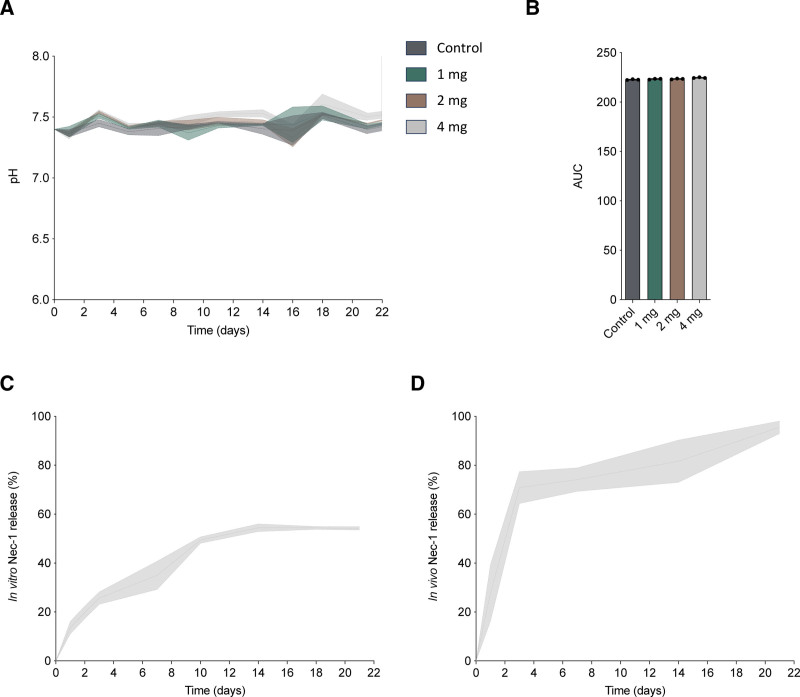
Nec-1 MPs exhibit sustained drug release with minimal pH impact. A, pH stability of Nec-1-loaded MPs (1, 2, and 4 mg) in CMRL culture medium during 21 d at 37 °C. B, AUC analysis of pH profiles. Cumulative release profile of Nec-1 (% of total encapsulated drug) from 4 mg MPs during 21 d (n = 3) in vitro (C) and in vivo (D). AUC, area under the curve; CMRL, Connaught Medical Research Laboratories; MP, microparticle; Nec-1, necrostatin-1. Created in BioRender.

In PBS, the initial pH was 7.4, which decreased to 6.69 (IQR, 6.48–6.52) by day 5, followed by stabilization at 6.5 for the remainder of the experiment (**Figure S1A, SDC,** https://links.lww.com/TXD/A857). This early acidification also occurred in the absence of MPs, suggesting that it is not solely due to PLGA hydrolysis. In contrast, CMRL media remained stable at ~7.4 (IQR, 7.34–7.53) throughout the experiment, likely due to its stronger intrinsic buffering capacity, including bicarbonate, (4-[2-hydroxyethyl]-1-piperazineethanesulfonic acid), and protein content (Figure 2A). Area under the curve analysis of the pH profiles revealed no significant differences between MP concentrations in either PBS (**Figure S1B, SDC,** https://links.lww.com/TXD/A857) or CMRL (Figure 2B), indicating that varying MP doses do not cause measurable long-term pH changes. These results suggest that Nec-1 MP degradation has minimal impact on sustained media pH and is likely safe for colocalization with islets.

To assess Nec-1 release kinetics in vitro, 4 mg of Nec-1 MPs were incubated in PBS at 37 °C and sampled during 21 d. Drug release was quantified by HPLC of both supernatant and residual MPs at each time point (Figure 2C). The release profile exhibited an initial burst, with a median of 24.83% released by day 3 (IQR, 21.81–30.34), followed by a gradual, sustained release until day 14, when the median cumulative release was 53.62% (IQR, 52.25–57.44), after which the release curve plateaued. By day 18, the median cumulative release was 54.14% (IQR, 53.46–55.45), and by day 21, the median was 53.51% (IQR, 53.49–55.51), indicating no significant increase between these time points. This plateau likely reflects PLGA matrix plasticization and reduced drug diffusivity within the polymer, consistent with water-induced polymer degradation and bulk erosion dynamics.^30,31^

Nec-1 release kinetics were next evaluated in vivo following subcapsular renal transplantation of Nec-1 MPs embedded in collagen. By day 1, median cumulative release reached 33.88% (IQR, 5.42–44.04) and increased to 73.56% by day 3 (IQR, 58.43–80.57; Figure 2D). Release progressed more gradually thereafter, with median cumulative values of 78.28% (IQR, 64.55–79.53) at day 7 and 87.78% (IQR, 64.51–92.54) at day 14. By day 21, cumulative release approached completion, with a median of 96.75% (IQR, 90.63–99.20). Compared with in vitro conditions, Nec-1 MPs exhibited faster and more complete drug release in vivo, consistent with enhanced polymer hydration and degradation in the physiological environment.

Together, these data demonstrate that Nec-1 MPs provide sustained drug delivery while maintaining pH stability, supporting their suitability for localized islet cotransplantation and enabling determination of an effective working concentration of Nec-1 for subsequent studies.

### Nec-1 MPs Protect Islets From ER Stress-induced Cell Death and Limit Stress-induced Cell Death and Preserve Glucose-stimulated Insulin Secretion

To evaluate the protective effect of Nec-1 MPs on islet viability and function under ER stress, isolated mouse islets were coincubated for 24 h with 4 mg of Nec-1-loaded MPs (containing 14.36 μg (IQR, 12.28–15.84 μg) of Nec-1), which corresponds to a release of 3.59 μg (IQR, 3.07–3.96 μg) during 24 h and an effective working concentration of 2.87 μM (IQR, 2.45–3.17 μM), or with empty MPs. Following this, islets were exposed to 5 μM Thp for an additional 24 h. Thp is a sarco/ER Ca2^+^-ATPase inhibitor that induces ER stress by disrupting calcium homeostasis, thereby impairing β-cell function and insulin secretion. Four experimental groups were assessed: empty MP, empty MP+Thp, Nec-1 MPs, and Nec-1 MPs+Thp. As a positive control, islets were pretreated with 100 μM soluble Nec-1 before Thp exposure. This served as a benchmark for comparison with MP-delivered Nec-1.

**FIGURE 3.**
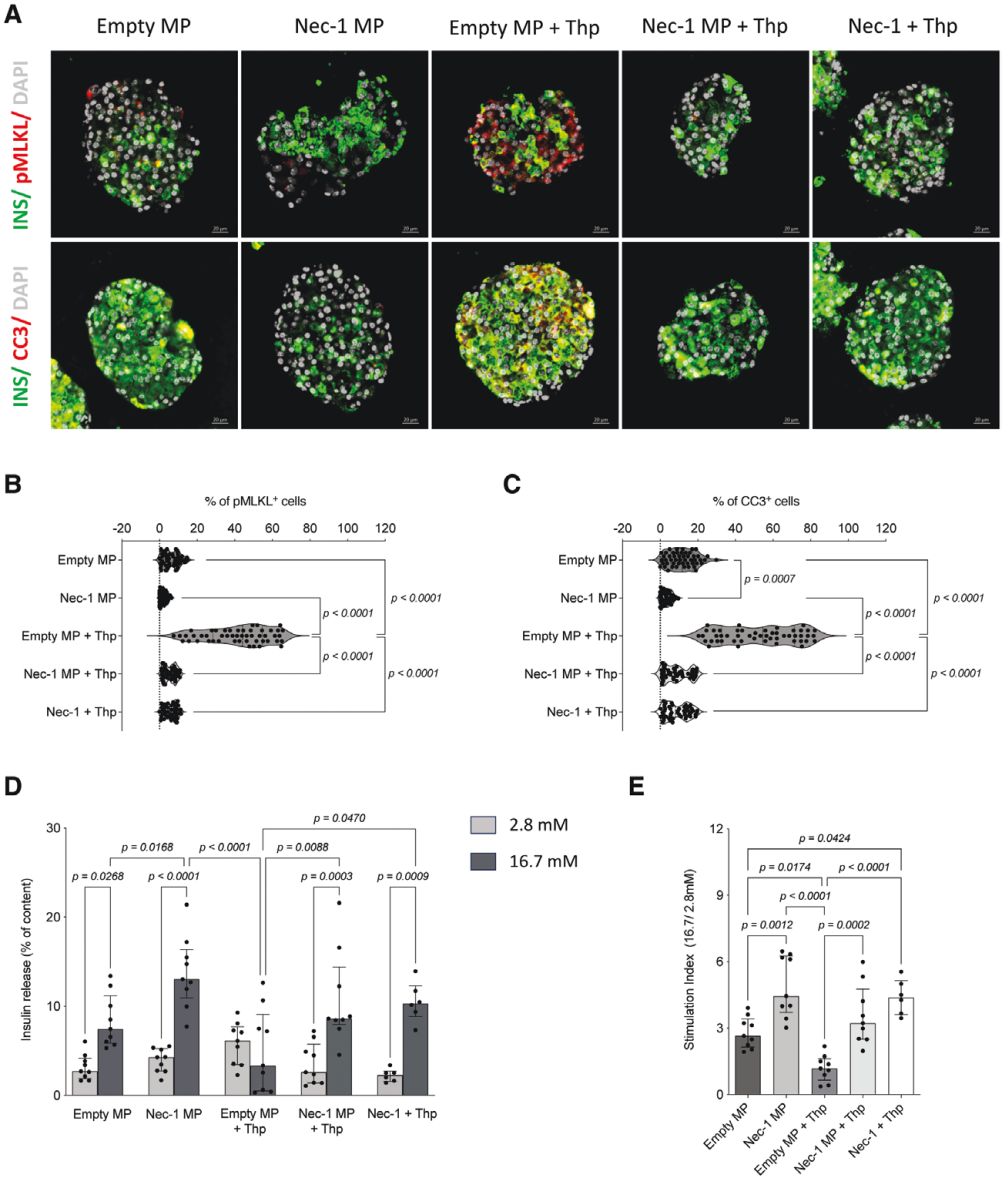
Nec-1 MPs preserve glucose-stimulated insulin secretion under ER stress conditions. A, Insulin release, expressed as a percentage of total content, in response to low and high glucose concentrations in the absence or presence of Thp. B, SI calculated as the ratio of insulin release at high vs low glucose. N = 10 independent replicates per group. Between-group comparisons were performed using the Kruskal-Wallis test with Dunn’s post hoc test for multiple comparisons. CC3, Cleaved caspase 3; ER, endoplasmic reticulum; MP, microparticle; Nec-1, necrostatin-1; pMLKL, phosphorylated mixed lineage kinase domain-like protein; SI, Stimulation Index; Thp, thapsigargin. Created in BioRender.

To assess Nec-1 MP protection, we quantified the number of pMLKL^+^ and CC3^+^ cells (Figure 3A). pMLKL staining showed minimal necroptotic activity in the empty MP (5.41% [IQR, 3.14%–9.67%]) and Nec-1 MP groups (2.15% [IQR, 0.46%–3.47%]; Figure 3B). Exposure to Thp significantly increased pMLKL^+^ cells in the empty MP+Thp group (44.15% [IQR, 28.33%–54.63%]) compared with empty MP alone (*P* < 0.0001), whereas cotreatment with Nec-1 MPs substantially attenuated this response (5.98% [IQR, 2.69%–8.33%]) to levels comparable with empty MP and Nec-1 MP groups. There were no differences between Nec-1 MPs+Thp and soluble Nec-1+Thp (6.28% [IQR, 2.60%–8.67%]). Notably, the empty MP+Thp group exhibited significantly higher pMLKL^+^ cell numbers than all other groups (*P *< 0.0001). CC3 staining revealed low baseline apoptosis in Empty MP islets (11.66% [IQR, 5.58%–17.37%]; Figure 3C); however, the percentage of CC3^+^ cells was significantly lower in the Nec-1 MP group (3.15% [IQR, 1.02%–5.50%]; *P = *0.0007). Exposure to Thp markedly increased CC3^+^ cells in the empty MP+Thp group (54.61% [IQR, 32.62%–71.39%]) compared with empty MP (*P* < 0.0001), whereas Thp cotreatment with Nec-1 MPs significantly mitigated this effect (8.03% [IQR, 2.64%–15.93%]; *P *< 0.0001), restoring apoptosis to baseline levels. There were no differences between Nec-1 MPs+Thp and soluble Nec-1+Thp (8.04% [IQR, 2.80%–14.08%]). Importantly, the empty MP+Thp group exhibited significantly higher CC3^+^ cell numbers than all other experimental conditions (*P *< 0.0001).

As shown in Figure 3D, islets treated with empty MP exhibited a clear glucose-stimulated insulin response, with insulin release increasing from a median of 2.72% (IQR, 1.91%–4.18%) under low glucose to 7.46% (IQR, 5.87%–11.18%) under high glucose (*P = *0.0318). Treatment with Nec-1 MPs alone further enhanced glucose responsiveness, with insulin release rising from 4.30% (IQR, 2.76%–5.26%) to 13.04% (IQR, 10.96%–16.35%; *P *< 0.0001), which was significantly higher than islets treated with empty MP under high glucose (*P = *0.0209). In contrast, Thp exposure abolished glucose-stimulated insulin secretion (GSIS), as islets in the empty MP+Thp group secreted 6.15% (IQR, 3.48%–7.69%) under low glucose and 3.36% (IQR, 0.53%–9.08%) under high glucose, confirming ER stress-induced β-cell dysfunction. Notably, cotreatment with Nec-1 MPs preserved glucose responsiveness, with insulin release increasing from 2.63% (IQR, 1.42%–5.74%) to 8.63% (IQR, 7.97%–14.42%) in the Nec-1 MPs+Thp group (*P = *0.0006), which was significantly higher than the empty+Thp condition (*P = *0.0117).

Consistent with these findings, the Stimulation Index (SI; Figure 3E) decreased markedly in the empty MP+Thp group (1.18; IQR, 0.65–1.62) compared with empty MP islets (2.65; IQR, 2.14–3.42; *P = *0.0158). Nec-1 MPs alone significantly increased the SI compared with control empty MP (4.45; IQR, 3.71–6.27; *P = *0.0013), demonstrating enhanced glucose responsiveness. Under ER stress, Nec-1 MPs also significantly improved SI relative to empty MP+Thp (3.23; IQR, 2.51–4.77; *P = *0.0002). Although the SI in Nec-1 MPs+Thp-treated islets was slightly lower than Nec-1 MPs alone, the difference was not statistically significant, indicating that Nec-1 delivery effectively preserves β-cell function under ER stress and maintains glucose responsiveness near baseline levels. Notably, Nec-1 delivered via MPs elicited protective effects comparable with those observed with 100 μM soluble Nec-1, with no significant differences in GSIS (low: 2.27% [IQR, 1.59%–2.74%]; high: 10.31% [IQR, 8.87%–12.28%]) or SI (4.38 [IQR, 3.60–5.14]), despite substantially lower effective drug exposure.

Collectively, these results show that sustained Nec-1 delivery from MPs effectively preserves islet viability and function under ER stress, maintaining glucose responsiveness comparable with pharmacologic soluble Nec-1 despite markedly reduced drug exposure.

### Nec-1 MPs Mitigate ER Stress-induced Inflammatory Cytokine Release

**FIGURE 4.**
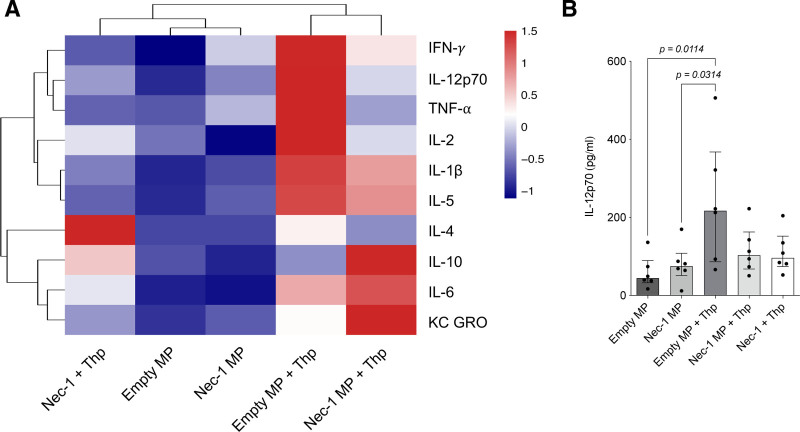
Nec-1 MPs mitigate ER stress-induced inflammatory cytokine release. A, Heatmap representation showing levels of 10 proinflammatory cytokines in culture supernatants collected after coincubation of mouse islets with 4 mg Nec-1-loaded or empty MPs, followed by exposure to 5 μM Thp for induction of ER stress. As a positive control, islets were pretreated with 100 μM soluble Nec-1 before Thp exposure. B, Quantification of IL-12p70 levels in the culture medium under the same conditions. N = 6 independent replicates per group. Between-group comparisons were performed using the Kruskal-Wallis test with Dunn’s post hoc test for multiple comparisons. ER, endoplasmic reticulum; IFN-γ, interferon-gamma; IL, interleukin; KC GRO, keratinocyte chemoattractant/growth-regulated oncogene; MP, microparticle; Nec-1, necrostatin-1; SI, Stimulation Index; Thp, thapsigargin; TNF-α, tumor necrosis factor alpha. Created in BioRender.

Culture supernatants from each experimental group were analyzed using the V-PLEX Proinflammatory Panel 1 Mouse Kit to quantify the secretion of inflammatory cytokines in response to ER stress induction. Clustering analysis of cytokine secretion profiles (Figure 4A) was performed using the median values from 6 independent replicates per group. The empty MP and Nec-1 MP groups exhibited low levels of proinflammatory cytokines and clustered closely together, indicating similar secretion profiles under non-Thp-treated conditions. In contrast, MP containing Thp-treated samples (empty MP+Thp and Nec-1 MP+Thp) formed a separate cluster, reflecting a distinct, inflammation-associated cytokine pattern. Interestingly, soluble Nec-1+Thp clustered independently from any other group, but its inflammatory profile was more like that of empty MP and Nec-1 MP groups than empty MP+Thp and Nec-1 MP+Thp groups.

Thp treatment in the empty MP condition induced a robust upregulation of multiple cytokines, including IFN-γ, IL-1β, IL-2, IL-4, IL-5, IL-6, IL-10, IL-12P70, KC GRO, and TNF-α, consistent with ER stress-mediated inflammation. Importantly, Thp treatment in the presence of Nec-1 MPs resulted in only a modest increase in cytokine secretion compared with untreated conditions, and cytokine levels remained lower than those observed in the empty MP+Thp group, suggesting that Nec-1 MPs mitigated the Thp-induced inflammatory response.

Although the heatmap illustrates differential cytokine profiles, statistical analysis showed no significant differences between treatment groups for most cytokines, except IL-12p70 (Figure 4B). Under Empty MP conditions, IL-12p70 secretion was relatively low, with a median of 44.37 pg/mL (IQR, 32.41–89.36 pg/mL). Treatment with Nec-1 MPs slightly increased IL-12p70 levels, yielding a median of 74.85 pg/mL (IQR, 50.99–107.9 pg/mL), but this change was not statistically significant. Addition of Thp to the Empty MP group significantly elevated IL-12p70 secretion, with a median of 217.1 pg/mL (IQR, 86.20–367.6 pg/mL; *P* = 0.0381), consistent with ER stress-induced inflammation. In contrast, Thp treatment in the presence of Nec-1 MPs resulted in a more modest increase, with a median of 103.3 pg/mL (IQR, 67.72–162.4 pg/mL), and did not differ significantly from Nec-1 MP alone. No significant differences were observed in IL-12p70 secretion between Nec-1 MP+Thp and Nec-1+Thp groups. Together, these results indicate that Thp strongly upregulates IL-12p70 secretion, whereas Nec-1 MPs partially attenuate this ER stress-induced cytokine response. Secretion profiles of IFN-γ, IL-1β, IL-2, IL-4, IL-5, IL-6, IL-10, KC GRO, and TNF-α can be found in **Figure S2A–I (SDC,** https://links.lww.com/TXD/A857).

## DISCUSSION

Early β-cell loss remains a critical barrier in ITx, particularly during the first days posttransplant, when inflammatory, ischemic, and ER stressors contribute to substantial cell death. Both apoptosis and necrosis are well-recognized mechanisms underlying this loss, and while several pharmacological strategies exist to inhibit apoptosis, effective interventions for necrosis are lacking due to its largely unregulated nature.^12-15^ Necroptosis, a regulated form of necrotic cell death driven by Receptor Interacting Protein Kinases (RIPK) 1, RIPK3, and MLKL, therefore represents a pharmacologically actionable therapeutic target.^32^ Necroptotic cell death not only reduces viable β-cell mass but also amplifies local inflammation through the release of damage-associated molecular patterns, exacerbating graft injury.^33^ This study builds on our previous work, in which pretreatment of human islets with Nec-1 for 24 h before transplant led to improved survival and enhanced euglycemic rates in a marginal mass syngeneic transplant model, demonstrating proof of concept for RIPK1 inhibition. Herein, we have developed a localized, sustained-release system using biodegradable PLGA MPs to deliver Nec-1 directly to the transplant microenvironment, with the goal of extending this protective effect into the critical early posttransplant period. This approach ensures continuous, localized exposure to Nec-1 while providing improved control over dosing kinetics and minimizing systemic exposure.

PLGA MPs are clinically validated carriers for controlled drug delivery, with tunable degradation kinetics and minimal perturbation of the local microenvironment.^21-23^ The Nec-1 MPs developed in this study exhibited favorable physicochemical properties, and sustained-release kinetics aligned with the window of greatest vulnerability for transplanted β-cells.^2^ In vivo, drug release was faster and more complete than in vitro, likely reflecting enhanced polymer hydration and degradation under physiological conditions, facilitating bioavailable drug delivery directly at the transplant site.^34^ Importantly, the MPs maintained media pH, mitigating concerns of local acidosis that could compromise islet viability.

Mechanistically, Nec-1 MPs reduced Thp-induced pMLKL and CC3 expression, demonstrating protection of β-cells from stress-induced cell death. These findings indicate that Nec-1 exerts pleiotropic effects, modulating both necroptotic and apoptotic pathways and broader RIPK1-dependent stress responses.^35-41^ Functionally, Nec-1 MPs preserved GSIS under Thp-induced ER stress, maintaining stimulation indices comparable with those observed under nonstress conditions. GSIS assays were performed in minimal, glucose-controlled RPMI 1640 (without added proteins or growth factors), ensuring preserved glucose responsiveness reflects genuine β-cell function rather than nutrient-driven artifacts. These results suggest that MP-mediated Nec-1 delivery effectively inhibits RIPK1-mediated necroptosis while maintaining biocompatibility. Although complementary assessments such as C-peptide secretion or intracellular calcium flux analyses could provide additional insight, GSIS under defined minimal conditions offers a robust and physiologically relevant measure of β-cell function.

Nec-1 MPs also attenuated Thp-induced secretion of IL-12p70, demonstrating a modest anti-inflammatory effect. No other proinflammatory cytokines were significantly reduced, and therefore, the IL-12p70 effect should not be interpreted as broad inflammatory suppression. Fetal bovine serum present in culture medium contains basal levels of cytokines, including interleukins, interferons, and TNF family members; hence, removing fetal bovine serum could increase cytokine assay sensitivity and reveal clearer treatment differences. Beyond direct β-cell protection, localized Nec-1 delivery may preserve intra-islet macrophages and passenger leukocytes, which are increasingly recognized as mediators of β-cell health and glucose responsiveness.^42,43^ Preserving these cells could support the maintenance of GSIS and promote a more immunoregulatory graft environment. Furthermore, conditioning of passenger leukocytes via extracellular vesicle signaling may provide an additional layer of immune modulation. For example, Sullivan et al^44^ demonstrated that exosomes can coat bystander lymphocytes with IL-35, inducing exhaustion and secondary suppression in non–regulatory T cells. Such localized immune conditioning could complement the cytoprotective effects of Nec-1, collectively enhancing graft survival and functional integration.

Our findings align with ongoing strategies to improve islet graft survival via localized interventions, such as hydrogels, MPs, and encapsulation systems delivering antiapoptotic, antioxidant, or provascularization agents.^45,46^ Although these approaches have shown promise in mitigating early β-cell loss, necroptosis-targeted interventions remain relatively unexplored. Localized Nec-1 delivery offers the advantage of minimizing systemic exposure and off-target effects while providing sustained protection during the period of highest vulnerability. The relatively low total dose of Nec-1 delivered by MPs is sufficient to confer functional protection, highlighting the efficiency of localized, intra-islet drug delivery. This strategy is particularly relevant for stem cell-derived islets, where enhanced survival could reduce the number of cells required per transplant, lowering production costs and facilitating broader clinical application.

Although limited to in vitro validation, the in vitro findings, together with in vivo release data, provide a foundation for future evaluation in physiologically relevant transplantation models, such as portal vein or subcutaneous transplantation, where inflammatory and ischemic stress are more pronounced. Mechanistic studies, including macrophage depletion and add-back experiments, will help distinguish β-cell-intrinsic from macrophage-mediated effects. Incorporating sex as a biological variable is also essential, given the known differences in STZ susceptibility and β-cell resilience between male and female mice, as well as potential sex-dependent differences in the maturation of SC-islets^47^ and xenoislets.^48^ Our rationale for using first-generation Nec-1 is supported by its pleiotropic protective effects in islets, which extend beyond canonical necroptosis inhibition to include apoptosis modulation, inflammatory attenuation, and metabolic stress protection. Future work may involve next-generation RIPK1 inhibitors with improved specificity, such as Nec-1s or GSK2982772, or combination therapies targeting multiple cell death pathways, including RIPK3, MLKL, and apoptotic signaling. Integrating these strategies into localized delivery platforms may yield additive or synergistic protection.

Overall, this study demonstrates that localized, sustained Nec-1 delivery using PLGA MPs can protect islets from ER stress-induced cell death, preserve glucose responsiveness, and limit inflammatory signaling at low total drug doses. By minimizing systemic exposure while extending protection through the early posttransplant period, this strategy represents a translationally relevant approach to improve islet engraftment, with applicability to stem cell-derived and xenogeneic islet replacement therapies. By reducing islet requirements and expanding transplantation eligibility, Nec-1 MPs could improve outcomes for patients with type 1 diabetes and support broader clinical translation.

## ACKNOWLEDGEMENTS

The University of Alberta is situated on Treaty 6 territory, the traditional lands of First Nations and Métis people.

## Supplementary Material


