# Supplementary material for: In Vitro Evaluation of Necrostatin-1 Microparticles for Limiting Stress-induced Cell Death and Preserving Islet Function
Source: Transplant Direct. 2026 Apr 23;12(5):e1945. doi: 10.1097/TXD.0000000000001945 (PMC13108664; doi:10.1097/TXD.0000000000001945)
Supplement: Supplementary file 1 [file txd-12-e1945-s001.pdf]

Supporting information for

In Vitro Evaluation of Necrostatin-1 Microparticles for Enhancing Islet Viability and Function

Cuesta-Gomez *et al.*

*Corresponding author. Email: apepper@ualberta.ca

This PDF file includes:

Figs. S1 to S2

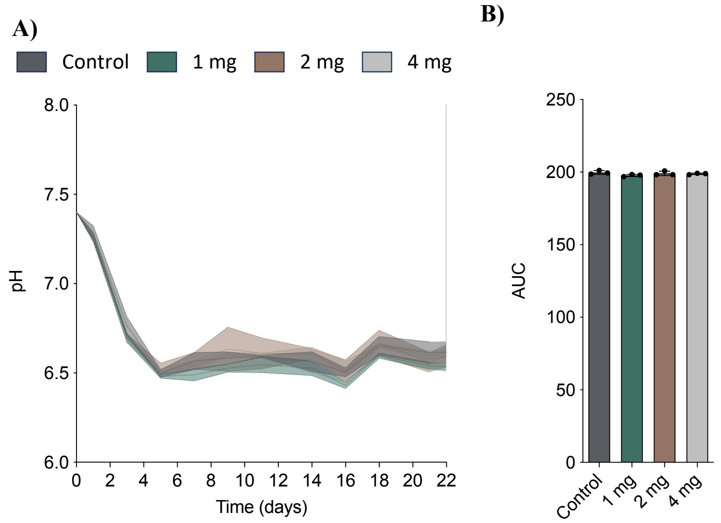

Figure S1. Necrostatin-1 microparticles exhibit sustained drug release with minimal pH impact. A) pH stability of Nec-1-loaded MPs (1, 2, and 4 mg) in PBS over 21 days at 37°C. B) Area under the curve (AUC) analysis of pH profiles.

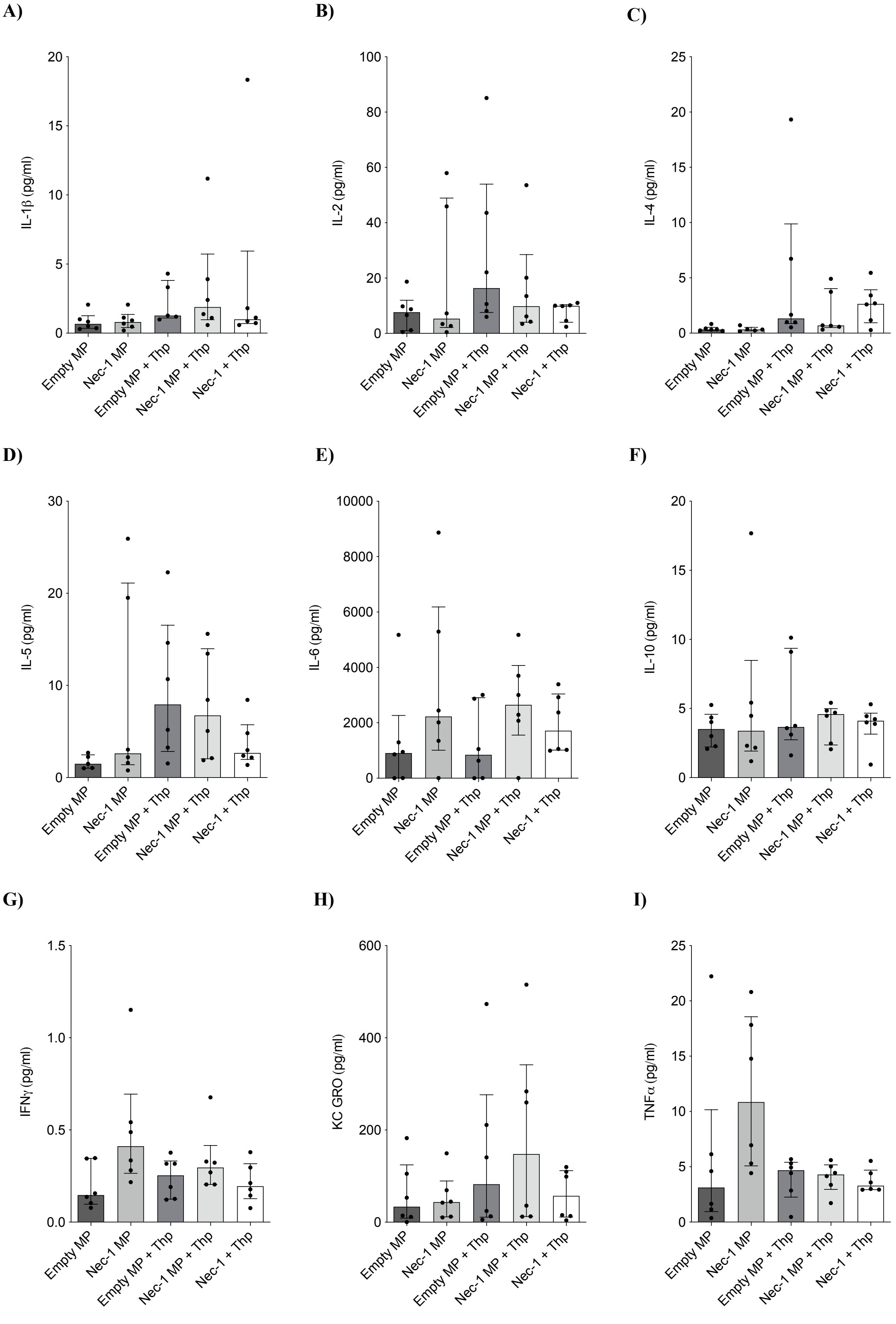

A)
B)
C)
D)
E)
F)
G)
H)
I)
IL-1β (pg/ml)
IL-2 (pg/ml)
IL-4 (pg/ml)
IL-5 (pg/ml)
IL-6 (pg/ml)
IL-10 (pg/ml)
IFNγ (pg/ml)
KC GRO (pg/ml)
TNFα (pg/ml)
0
5
10
15
20
25
30
40
60
80
100
200
400
600
2000
4000
6000
8000
10000
0.0
0.5
1.0
1.5
Empty MP
Nec-1 MP
Empty MP + Thp
Nec-1 MP + Thp
Nec-1 + Thp

Figure S2. Necrostatin-1 microparticles mitigate ER stress-induced inflammatory cytokine release. A) Quantification of L-1β, B) IL-2, C) IL-4, D) IL-5, E) IL-6, F) IL-10, G) IFN-γ, H) KC/GRO, I) TNF-α in culture supernatants collected 24 hours after co-incubation of mouse islets with 4 mg Nec-1–loaded or empty microparticles (MPs), followed by 24-hour exposure to 5 μM thapsigargin (Thp) to induce ER stress. N=6 independent replicates per group. Between-group comparisons were performed using the Kruskal-Wallis test with Dunn's post-hoc test for multiple comparisons.
